# Isothiocyanates, Nitriles, and Epithionitriles from Glucosinolates Are Affected by Genotype and Developmental Stage in *Brassica oleracea* Varieties

**DOI:** 10.3389/fpls.2017.01095

**Published:** 2017-06-22

**Authors:** Franziska S. Hanschen, Monika Schreiner

**Affiliations:** Leibniz Institute of Vegetable and Ornamental CropsGrossbeeren, Germany

**Keywords:** glucosinolate, isothiocyanate, epithionitriles, nitrile, ontogeny, broccoli, cauliflower, cabbage

## Abstract

Vegetables of the *Brassica oleracea* group, such as broccoli, cauliflower, and cabbage, play an important role for glucosinolate consumption in the human diet. Upon maceration of the vegetable tissue, glucosinolates are degraded enzymatically to form volatile isothiocyanates, nitriles, and epithionitriles. However, only the uptake of isothiocyanates is linked to the cancer-preventive effects. Thus, it is of great interest to evaluate especially the isothiocyanate formation. Here, we studied the formation of glucosinolates and their respective hydrolysis products in sprouts and fully developed vegetable heads of different genotypes of the five *B. oleracea* varieties: broccoli, cauliflower as well as white, red, and savoy cabbages. Further, the effect of ontogeny (developmental stages) during the head development on the formation of glucosinolates and their respective hydrolysis products was evaluated at three different developmental stages (mini, fully developed, and over-mature head). Broccoli and red cabbage were mainly rich in 4-(methylsulfinyl)butyl glucosinolate (glucoraphanin), whereas cauliflower, savoy cabbage and white cabbage contained mainly 2-propenyl (sinigrin) and 3-(methylsulfinyl)propyl glucosinolate (glucoiberin). Upon hydrolysis, epithionitriles or nitriles were often observed to be the main hydrolysis products, with 1-cyano-2,3-epithiopropane being most abundant with up to 5.7 μmol/g fresh weight in white cabbage sprouts. Notably, sprouts often contained more than 10 times more glucosinolates or their hydrolysis products compared to fully developed vegetables. Moreover, during head development, both glucosinolate concentrations as well as hydrolysis product concentrations changed and mini heads contained the highest isothiocyanate concentrations. Thus, from a cancer-preventive point of view, consumption of mini heads of the *B. oleracea* varieties is recommended.

## Introduction

With a worldwide production of more than 71 million tons in 2013, *Brassica oleracea* vegetables, such as broccoli, cauliflower, and various cabbages, make up a substantial part of the human diet (FAOSTAT, 2016). *Brassica* vegetables are characterized by a certain group of sulfur-containing secondary plant metabolites – the glucosinolates (GLSs). GLSs are sulfur-containing secondary plant metabolites that can be classified according to the structure of their variable side chain into more than 130 aliphatic, aromatic, and indole GLSs (Agerbirk and Olsen, 2012). In the plant, GLSs play important roles in the plant’s defense against biotic stressors in general and act as effective deterrents against a multitude of pathogens in particular (Witzel et al., 2013). When cells are disrupted, GLSs that are stored in the plant vacuole or in specialized S-cells (Jørgensen et al., 2015) encounter endogenous myrosinases, β-D-thioglucosidases, that cleave D-glucose resulting in various hydrolysis products, such as isothiocyanates (ITCs), nitriles, or epithionitriles (EPTs), being released (Kissen et al., 2009). This enzymatic breakdown is of great importance for both food quality and also human health since it is the formed ITCs that are responsible for the sharp taste of mustard, radish, or broccoli sprouts (Halkier and Gershenzon, 2006) as well as for cancer preventing effects (Veeranki et al., 2015). Among other ITCs, 4-(methylsulfinyl)butyl ITC (sulforaphane, 4MSOB-ITC) from broccoli was identified to be a pleiotropic protective agent that interacts with multiple pathways of carcinogenesis (Zhang, 2012). For example, it interferes with the xenobiotic metabolism by inducing phase II enzymes (Boddupalli et al., 2012; James et al., 2012) as well as triggers cytostasis (Fimognari et al., 2002; Mi et al., 2007), and/or apoptosis (Xiao et al., 2006; Yeh and Yen, 2009).

**FIGURE 1:**
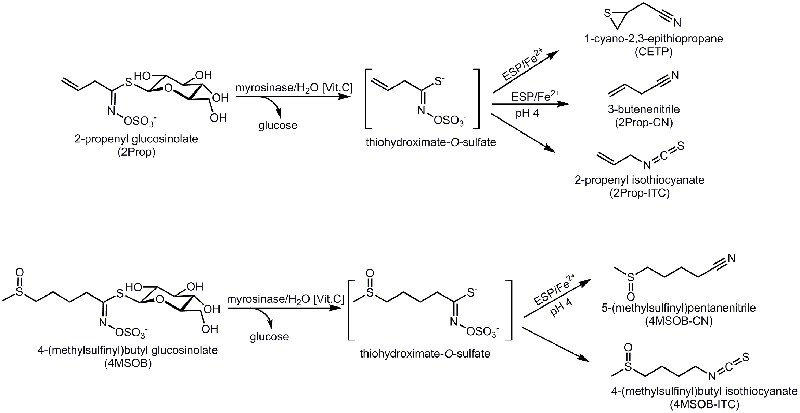
Enzymatic hydrolysis pathways of 2-propenyl glucosinolate (2Prop) and 4-(methylsulfinyl)butyl glucosinolate (4MSOB). ESP, epithiospecifier protein. The glucosinolate hydrolysis is started by myrosinase induced degradation with vitamin C being a cofactor for this enzyme. The resulting thiohydroximate-*O*-sulfate intermediate either spontaneously degrades to the isothiocyanate (ITC) and under acidic conditions to the nitrile. In presence of ESP, which is dependent on Fe^2+^, the formation of the epithionitrile from the alkenyl intermediate (2Prop) or of the nitrile from the non-alkenyl intermediate (4MSOB) is catalyzed.

However, in addition to myrosinases, among other Brassicales plants *Brassica* vegetables also often contain epithiospecifier proteins (ESPs) that affect the hydrolysis of GLS and this can result in nitriles or EPTs rather than ITCs being released (Tookey, 1973; Lambrix et al., 2001; Matusheski et al., 2006). In detail, after myrosinase cleaves D-glucose from the GLS, an unstable aglucone, the thiohydroximate-*O*-sulfate, is released (**Figure 1**). While in the absence of ESPs, the aglucones spontaneously rearrange to form ITCs and small amounts of nitriles, the degradation of the aglucones will be modified if ESP is present and EPTs are released from alkenyl GLSs. From non-alkenyl GLSs, in the presence of ESP the formation of nitriles is favored instead of ITC (**Figure 1**) (Burow and Wittstock, 2009; Wittstock and Burow, 2010). Notably, in *Arabidopsis thaliana* also nitrile specifier proteins (NSPs), that favor nitriles as well as epithiospecifier modifier (ESM), that favors isothiocyanate formation, have been reported (Zhang et al., 2006; Burow et al., 2009).

Since nitriles and EPTs were shown to have less health beneficial potential compared to ITCs (Shofran et al., 1998; Matusheski and Jeffery, 2001; Hanschen et al., 2015), it is of great interest to provide food rich in ITCs in respect to a healthy human diet.

With regard to GLS consumption, *B. oleracea* vegetables, among them broccoli (*B. oleracea* var. *italica*) and cauliflower (*B. oleracea* var. *botrytis*), are the most important source for GLS intake in Germany (Steinbrecher and Linseisen, 2009). With regard to the GLS present in these vegetables, broccoli contains mainly 4-(methylsulfinyl)butyl GLS (4MSOB; glucoraphanin) (Schonhof et al., 2004; Gu et al., 2014) and indol-3-ylmethyl GLS (I3M; glucobrassicin) (Schonhof et al., 2004; Steinbrecher and Linseisen, 2009). White cauliflower contains mainly 2-propenyl GLS (2Prop; sinigrin), 3-(methylsulfinyl)propyl GLS (3MSOP; glucoiberin), and I3M (Ciska et al., 2000; Schonhof et al., 2004; Steinbrecher and Linseisen, 2009). White (*B. oleracea* var. *capitata* f. *alba*), red (*B. oleracea* var. *capitata* f. *rubra*), and savoy cabbage (*B. oleracea* var. *sabauda*) are all rich in 2Prop and I3M. Further, white and savoy cabbage are also rich in 3MSOP, while red cabbage contains high levels of 4MSOB (Ciska et al., 2000).

Profiles and contents of GLSs in plants are affected by a number of ecophysiological factors, such as temperature, irradiation, nutrition, and water supply (Verkerk et al., 2009), by wounding and biotic factors such as herbivores (Mumm et al., 2008; Textor and Gershenzon, 2009), as well as intrinsic plant-determined factors, such as (onto)genetic influences that can cause an enormous variation in GLS levels even within the plant (Brown et al., 2003). Moreover, ontogenetic changes can lead to fluctuating GLS profiles depending on the stage of plant development. For example, while seeds usually have the highest concentrations of GLSs with aliphatic or aromatic GLSs being dominant (Brown et al., 2003; Bellostas et al., 2007; Pérez-Balibrea et al., 2010), after germination, GLS contents first decrease, but then later increase, with indole GLS contents being considerably higher (Brown et al., 2003; Bellostas et al., 2007; Wiesner et al., 2013b). Finally, GLSs also accumulate in the inflorescences and siliques containing the seeds (Brown et al., 2003; Bellostas et al., 2007). However, the genotype of *B. oleracea* vegetables is also known to influence the profile and concentrations of GLSs (Cartea et al., 2008; Gu et al., 2015) and the genotype can have a greater effect than environmental influences (Farnham et al., 2004).

To date, while much is known about GLSs in *B. oleracea* vegetables less is known with regard to the formation of GLS hydrolysis products in these vegetables and often literature is out of date (Daxenbichler et al., 1977) or lacks information on the *B. oleracea* genotype analyzed (Cole, 1975, 1976). In addition, it is also often the case that only ITCs were analyzed, but not the nitriles or EPTs (Gerendás et al., 2008) or the study was limited to analyzing just one GLS and its hydrolysis products, even in the latest literature (Matusheski et al., 2004; Jones et al., 2010; Sarvan et al., 2017). Further, to the best of our knowledge, there is only one single study on the ontogenetic influence on the formation of GLS hydrolysis products and this is limited to one variety of *B. oleracea*, namely cauliflower (Cole, 1980). Furthermore, this study includes no data on initial individual GLS content related to the corresponding formation of their individual hydrolysis products.

In humans, ITCs, such as 4MSOB-ITC, not only have anti-carcinogenic properties, but also exert a multitude of health beneficial effects, including anti-microbial, anti-inflammatory, and anti-thrombotic effects (Singh and Singh, 2012; Ku and Bae, 2014; Veeranki et al., 2015). In sharp contrast, nitriles and EPTs were shown to have less health protective potential (Shofran et al., 1998; Matusheski and Jeffery, 2001; Hanschen et al., 2015). Thus, in respect to a healthy human diet, it would be ideal to provide *Brassica*-based food that is rich in ITCs, but that has low nitrile and EPT concentrations.

Recently we showed that ITC formation from *Brassica* vegetables can also be optimized due to ideal vegetable preparation conditions such as by acidifying the raw vegetable portion (Hanschen et al., 2017). Another strategy would be the selection of cultivars or developmental stages with distinct promotion to ITC formation.

Here, we provide comprehensive data on concentration and composition of GLSs and the formation of their hydrolysis products in respect to genotype and developmental stage in the five most diet-important head-forming *B. oleracea* varieties in Germany and hence very relevant for human glucosinolate consumption (Steinbrecher and Linseisen, 2009). Individual GLSs as well as their derived hydrolysis products were quantified in 16 *B. oleracea* cultivars and both sprouts and fully developed heads were analyzed. Further, to identify developmental stages with high ITC concentrations, the effect of ontogeny of the head development was also determined in one cultivar of each *B. oleracea* variety.

## Materials and Methods

### Chemicals

Benzonitrile (≥99.9%), 3-butenenitrile (2Prop-CN; ≥98%), 3-(methylthio)propyl ITC (3MTP-ITC; ≥98%), 2-propenyl ITC (2Prop-ITC; ≥99%), 4-pentenenitrile (3But-CN; ≥97%), 3-phenylpropanenitrile (2PE-CN; ≥99%), and 2-phenylethyl isothiocyanate (2PE-ITC; ≥99%) were purchased from Sigma–Aldrich Chemie GmbH (Steinheim, Germany). 1-Cyano-2,3-epithiopropane [CETP; ≥97.6% (by GC-MS)] was purchased from Taros Chemicals GmbH Co. KG (Dortmund, Germany). 3-Indoleacetonitrile (IAN) (≥98%) was acquired from Acros Organics (Fischer Scientific GmbH, Schwerte, Germany). 3-Butenyl ITC (3But-ITC; ≥95%) was obtained from TCI Deutschland GmbH (Eschborn, Germany). 3-(Methylsulfinyl)propyl ITC (3MSOP-ITC) and 4-(methylthio)butyl ITC (4MTB-ITC; ≥98%) were purchased from Santa Cruz Biotechnology (Heidelberg, Germany). 4-(Methylsulfinyl)butyl ITC (4MSOB-ITC) was bought from Enzo Life Sciences GmbH (Lörrach, Germany). (*R*)-5-Vinyloxazolidine-2-thione (*R*-OZT) was purchased from Biosynth AG (Staad, Switzerland). 4-Hydroxybenzyl GLS (≥99%) and methylene chloride (GC Ultra grade) was obtained from Carl Roth GmbH (Karlsruhe, Germany). Acetonitrile (Ultra Gradient HPLC grade) was procured from J.T. Baker (Deventer, The Netherlands). NaSO_4_ (≥99%) and methanol (>99.9) was purchased from VWR International GmbH (Darmstadt, Germany).

All solvents were of LC-MS or GC-MS grade, water was of Milli-Q quality.

### Plant Material and Field Experiment

Sixteen cultivars of five *B. oleracea* varieties were field-grown in the years 2014 and 2015. The selected cultivars of the *B. oleracea* varieties are current cultivars of the latest *B. oleracea* collection and were chosen due to their partly similar cultivation requirements hence being at best comparable. According to the ecophysiological requirements, inflorescence *B. oleracea* vegetables, such as broccoli (*B. oleracea* var. *italica*) and cauliflower (*B. oleracea* var. *botrytis*), were grown from mid-March to the end of June (spring/summer cultivation), whereas head-forming *B. oleracea* vegetables, such as, white cabbage (*B. oleracea* var. *capitata* f. *alba*) red cabbage (*B. oleracea* var. *capitata* f. *rubra*), and savoy cabbage (*B. oleracea* var. *sabauda)*, were cultivated from mid-June to October (summer/autumn cultivation) (see Supplementary Table 1).

To ensure a certain genotypic diverse range for each variety several cultivars were selected. Broccoli cv. Iron Man and cauliflower cv. Momentum were obtained from Monsanto Agrar Deutschland GmbH (Borken, Germany). Broccoli cv. Sirtaki, cauliflower cv. Baltimore, and savoy cabbage cvs. Emerald, Daphne, and Capriccio were purchased from Nickerson Zwaan GmbH (Gerwisch, Germany). Broccoli cv. Marathon, cauliflower cvs. Abeni and Graffiti, and white cabbage cv. Perfecta were obtained from Volmary GmbH (Niedergörsdorf, Germany). White cabbages cvs. Marcello and Tolsma and red cabbage cv. Redma were acquired from Rijk Zwaan Welver GmbH (Welver, Germany) and red cabbage cv. Integro was purchased from Bejo Samen GmbH (Sonsbeck, Germany). Red cabbage cv. Roodkop 2 was obtained from Quedlinburger Saatgut mbH (Aschersleben, Germany). Except for cv. Roodkop 2, all other cultivars are F1 hybrids.

Seeds were sown (broccoli and cauliflower 18.03.2014 and 31.03.2015; cabbages 18.06.2014 and 22.06.2015) in substrate (Einheitserde Classic, medium structure, pH value 5.9, Einheitserde Werkverband e.V., Germany) and grown in a greenhouse at the Leibniz Institute of Vegetable and Ornamental Crops in Großbeeren, Germany. After 7–9 days (same developmental stage), some of the sprouts were harvested. Remaining plants were transplanted into substrate-filled pot trays (7 cm diameter) for 3 weeks. At the three-leaf stage, plants were transferred into the field (16.04.2014 and 22.04.2015) (52°20′59.2″N 13°18′57.9″E) in a randomized block design in quintuplicate. The five randomized plots per cultivar were 9.6 m^2^ each and contained 36 plants with a row/plant distance of 45/50 cm. Plants were protected from insects by cultivation nets. Therefore, neither in 2014 or 2015 plants were affected by pathogens or insects. According to standard practice, fertilization prior to planting and 1 month after planting was given as 250 kg of nitrogen/ha. Water was given as needed throughout the growth period. Climatic data of the field experiment is given in Supplementary Table 2 and Figure 9. From all cultivars, the fully developed head was harvested. The exact harvest dates are given in Supplementary Table 1.

To test the influence of ontogeny, mini head (immature), fully developed head (commercial), and the over-mature developmental stages of selected cultivars for each *B. oleracea* variety were taken: cvs. Iron Man (broccoli), Abeni (cauliflower), Marcello (white cabbage), Redma (red cabbage), and Emerald (savoy cabbage) (see Supplementary Table 1). As reported previously (Krumbein et al., 2007), the developmental stages were classified according to the BBCH scale [uniform coding of phenologically similar growth stages of plant species (BBCH-Monograph, 1997)].

### Harvest and Sample Preparation

At harvest day, in the morning three replicates of sprouts were frozen in liquid nitrogen, freeze-dried, and ground to a fine powder. Additionally, fresh material was used for quantification of GLS hydrolysis products: 250 mg of sprouts (4–7 sprouts) were mixed with 500 μL of water and homogenized using a mixer mill (MM 400, RETSCH GmbH, Haan, Germany).

For the analysis of the heads, from each of the five randomized plots, in the morning five heads were harvested and made up the five replicates. Replicates immediately were bought to the nearby lab. Here from each head of a replicate, two slices opposite to each other were taken (triangular shape), mixed (sample size 150–500 g), immediately frozen at -50°C, lyophilized, and ground to a fine powder for GLS analysis. Dry weights (DW) were determined from these freeze-dried samples in order to later calculate the amounts in the fresh weight (FW). For analysis of GLS hydrolysis products, 10 slices (neighboring to the slices for the GLS analysis, sample size 150–500 g) from the five vegetable heads (two eights of each head) were cut into 7 mm × 7 mm pieces and mixed. From this mixed sample, an aliquot (20 g) was homogenized as reported previously (Hanschen et al., 2015).

### Analysis of Glucosinolates

To determine the profiles and concentrations of GLS, the sample preparation protocol based on that reported by Wiesner et al. (2013a) was used, while the UHPLC protocol was identical to Hanschen et al. (2015). Briefly, 20 mg of lyophilized and ground plant tissue were extracted trice using 70% methanol (at 70°C for 10 min with 750, 500, and 500 μL of 70% methanol in the three extractions) in the presence of 0.5 μmol 4-hydroxybenzyl GLS as internal standard. The combined extracts were loaded onto DEAE-Sephadex A-25 ion-exchanger columns, desulfated using 75 μL of aryl sulfatase, and desulfo-GLSs were eluted with 1 mL of water. Analysis of desulfo-GLSs was performed as described previously (Hanschen et al., 2015) using an UHPLC Agilent 1290 Infinity System (Agilent Technologies, Böblingen, Germany) and a gradient of water and acetonitrile, and quantified at 229 nm via the internal standard.

### Determination of Glucosinolate Hydrolysis Products after Incubation

The quantification of the enzymatically formed GLS hydrolysis products was performed based on the protocol of Hanschen et al. (2015). Briefly, aliquots of homogenized tissue (250 mg of sprouts or 500 mg of fully developed vegetables after 30 min of homogenization at room temperature) were extracted using 2 mL of methylene chloride in the presence of 0.2 μmol of the internal standard benzonitrile. After a centrifugation step and separation of the methylene chloride phase the material was re-extracted with another 2 mL of methylene chloride and after centrifugation the combined extracts were dried using anhydrous sodium sulfate, concentrated under nitrogen gas flow to 300 μL, transferred into a vial, and analyzed using an Agilent 7890 A Series GC System (Agilent Technologies) equipped with an Agilent 7683 Series Autosampler, an Agilent 7683B Series Injector and an Agilent 5975C inert XL MSD. After splitless injection of 1 μL of sample solution at 190°C, analytes were separated using a SGE BPX5 column 30 m × 0.25 mm × 0.25 μM (VWR International GmbH, Darmstadt, Germany), He as carrier gas (1.8 mL/min) and a temperature gradient starting at 35°C (for 3 min) rising with 9°C/min to 90°C (2 min hold) then to 110°C with 3°C/min, further increased to 210°C with 9°C/min, then to 223°C with 3°C/min, then to 230°C with 9°C/min and finally the GC was heated to 310°C with 35°C/min (6 min hold). The MSD parameters were as follows: transfer line 310°C, ion source 230°C, EI (70 eV), scan range 30–240 m/z. Compounds were identified by comparing their mass spectra and retention times with those of authentic standards and with literature data (Kjaer, 1963; Spencer and Daxenbichler, 1980) and quantitation was done as reported previously using the total ion current (TIC), the internal standard and the response factors of available standards or, if the standard was unavailable, a response factor equal to that of the chemically most similar compound was used (Witzel et al., 2015).

### Statistical Analysis

To investigate differences between developmental stages one-way analysis of variance (ANOVA) was performed. For the comparison of means, Tukey’s HSD test was applied using the STATISTICA version 12 software [StatSoft, Inc. (2013)]. All experiments were carried out in quintuplicate. Principle component analysis (PCA) was done using PAST software, version 3.15. Scores and loadings are given in Supplementary Table 4.

## Results

### Glucosinolates in *B. oleracea* Varieties

**FIGURE 2:**
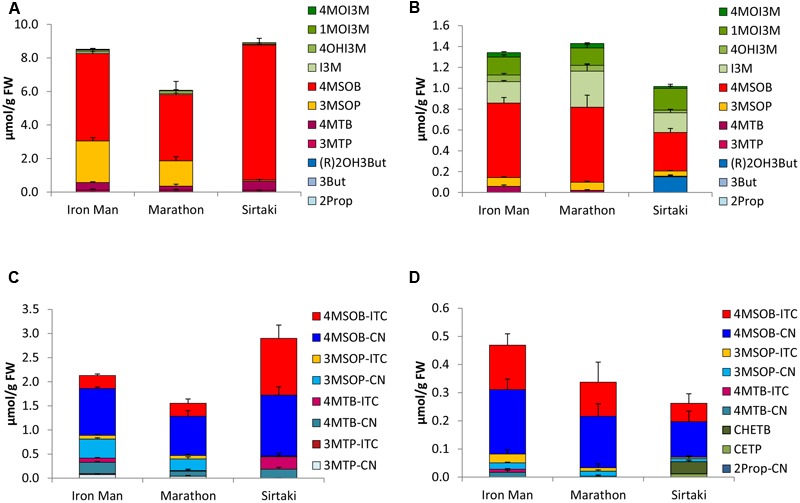
Glucosinolates (GLSs) [μmol/g fresh weight (FW)] and their hydrolysis products [μmol/g FW] in different cultivars of broccoli in sprouts [**(A)** GLSs, **(C)** hydrolysis products] and fully developed broccoli heads [**(B)** GLSs, **(D)** hydrolysis products]. Abbreviations: see **Table 2**.

**FIGURE 3:**
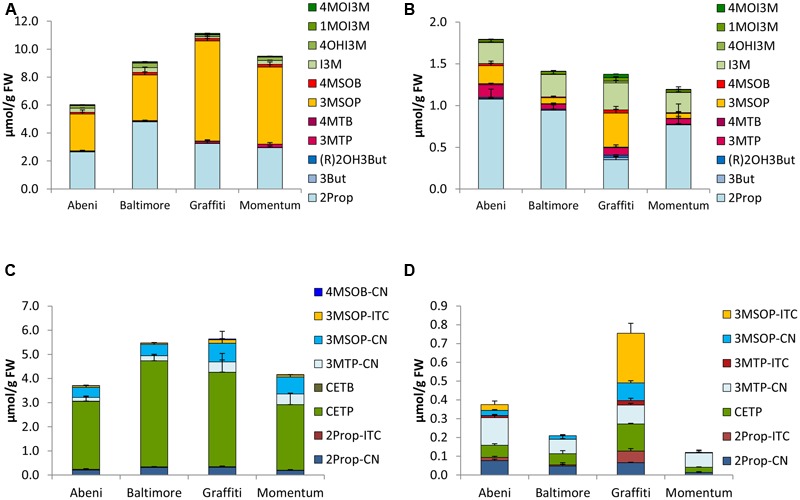
Glucosinolates [μmol/g FW] and their hydrolysis products [μmol/g FW] in different cultivars of cauliflower in sprouts [**(A)** GLSs, **(C)** hydrolysis products] and fully developed cauliflower heads [**(B)** GLSs, **(D)** hydrolysis products]. Abbreviations: see **Table 2**.

**FIGURE 4:**
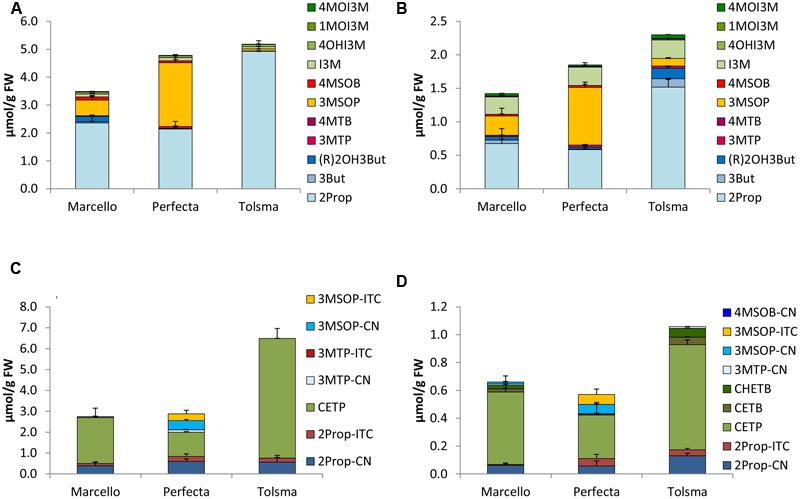
Glucosinolates [μmol/g FW] and their hydrolysis products [μmol/g FW] in different cultivars of white cabbage in sprouts [**(A)** GLSs, **(C)** hydrolysis products] and fully developed white cabbage heads [**(B)** GLSs, **(D)** hydrolysis products]. Abbreviations: see **Table 2**.

**FIGURE 5:**
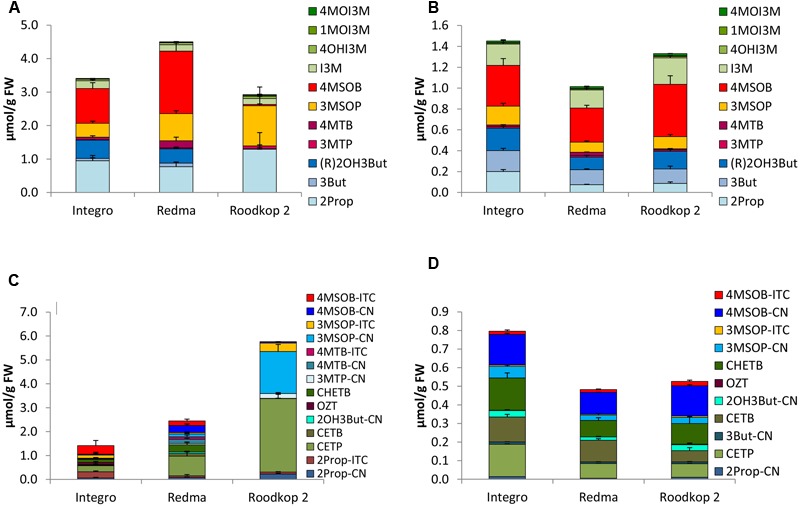
Glucosinolates [μmol/g FW] and their hydrolysis products [μmol/g FW] in different cultivars of red cabbage in sprouts [**(A)** GLSs, **(C)** hydrolysis products] and fully developed red cabbage heads [**(B)** GLSs, **(D)** hydrolysis products]. Abbreviations: see **Table 2**.

**FIGURE 6:**
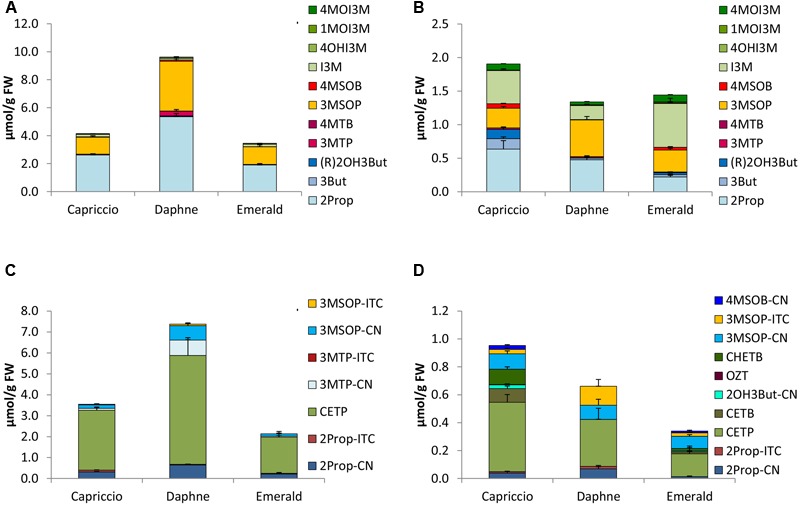
Glucosinolates [μmol/g FW] and their hydrolysis products [μmol/g FW] in different cultivars of savoy cabbage in sprouts [**(A)** GLS, **(C)** hydrolysis products] and fully developed savoy cabbage heads [**(B)** GLSs, **(D)** hydrolysis products]. Abbreviations: see **Table 2**.

In the five different *B. oleracea* varieties tested, a total of 13 different GLS were identified, namely the alkenyls 2Prop, 3-butenyl GLS (3But), and (*R*)-2-OH-3-butenyl GLS [(R)2OH3But], the side chain sulfur-containing aliphatic 3-(methylthio)propyl GLS (3MTP), 4-(methylthio)butyl GLS (4MTB), 3MSOP, 4MSOB, and 5-(methylsulfinyl)pentyl GLS, the aromatic 2-phenylethyl (2PE) GLS, and the indoles I3M; 4-hydroxyindol-3-ylmethyl GLS (4OHI3M), 1-methoxyindol-3-ylmethyl GLS (1MOI3M), and 4-methoxyindol-3-ylmethyl GLS (4MOI3M). The GLS profiles of the vegetables grown in 2014 and 2015 were often similar. Therefore, the results of 2014 are displayed in the **Figures 2–6**, while the results of 2015 are given as Supplementary Figures 1–5. In general, in these varieties, 2Prop, 3MSOP, 4MSOB, and I3M were most abundant. Sprouts always had higher GLS concentrations compared to the full head developmental stage (**Figures 2A,B–6A,B**). With 11.24 μmol/g FW, the highest total GLS content was found in cauliflower sprouts of cv. Graffiti (**Figure 3A**), whereas with 0.74 μmol/g FW, the lowest total GLS content was observed in fully developed cauliflower heads of cv. Momentum (Supplementary Figure 2B).

#### Broccoli

The main GLS of the broccoli cvs. Iron Man, Marathon, and Sirtaki was 4MSOB. Whilst cv. Sirtaki sprouts had the highest 4MSOB level (8.05 μmol/g FW), the corresponding fully developed head of this cultivar showed the lowest 4MSOB concentration (0.27 μmol/g FW) (**Figures 2A,B**). Except for cv. Sirtaki, broccoli sprouts were also quite rich in 3MSOP and to a lesser extent in 4MTB, while the fully developed heads also contained considerable concentrations of the indole GLS I3M and 1MOI3M (**Figure 2B**). Fully developed heads of cv. Sirtaki also showed considerable concentrations of (R)2OH3But (0.15 μmol/g FW) (**Figure 2B**).

#### Cauliflower

Cauliflower sprouts of the cvs. Abeni, Baltimore, Graffiti, and Momentum were rich in both 2Prop and 3MSOP (**Figure 3A**) and 2Prop was also a dominating GLS of the fully developed head. Further, the fully developed heads also exhibited high concentrations of I3M (**Figure 3B**). In sprouts of cv. Graffiti, in 2014, 3MSOP was the GLS with the highest concentration with 7.15 μmol/g FW (**Figure 3A**), while in 2015, 2Prop had the highest concentrations (Supplementary Figure 2A). Sprouts of cv. Abeni had the lowest total GLS concentration, while at the fully developed head stage, cv. Momentum showed the lowest GLS concentrations by trend (**Figures 3A,B**).

#### White Cabbage

Among the white cabbage cvs. Marcello, Perfecta, and Tolsma, 2Prop was one of the main GLSs in both developmental stages sprouts and fully developed heads (**Figures 4A,B**). Sprouts of cv. Tolsma always contained the highest concentrations of 2Prop (4.92 μmol/g FW). Further, in both sprouts and fully developed heads of cvs. Marcello and Perfecta were also rich in 3MSOP, and fully developed heads of all three different cultivars also contained considerable concentrations of I3M (**Figures 4A,B**).

#### Red Cabbage

In both sprouts and fully developed heads of red cabbage, 4MSOB was the predominant GLS (**Figures 5A,B**), except for sprouts of cv. Roodkop 2, which were nearly devoid of this GLS in 2014, but not in 2015 (**Figure 5A** and Supplementary Figure 4A). Sprouts of cv. Redma showed the highest 4MSOB concentration (1.87 μmol/g FW). Next to 4MSOB, red cabbage sprouts were also rich in 2Prop, (R)2OH3But, and 3MSOP (**Figure 5A**). Additionally, the fully developed heads contained 3But and I3M in higher levels (**Figure 5B**).

#### Savoy Cabbage

The major GLSs of sprouts of the savoy cabbage cvs. Capriccio, Daphne, and Emerald were 2Prop followed by 3MSOP (**Figure 6A**). In sprouts of cv. Daphne, these GLS were most abundant with 5.35 μmol of 2Prop and 3.56 μmol of 3MSOP. In fully developed heads, I3M was also present, which was the main GLS in cv. Emerald (0.65 μmol/g FW) (**Figure 6B**).

### Glucosinolate Hydrolysis Products in *B. oleracea* Varieties

Upon cell disruption of *B. oleracea* several volatile hydrolysis products were formed that were quantified by GC-MS analysis. In the five *B. oleracea* varieties analyzed, a total of 22 GLS hydrolysis products were identified, among them 11 nitriles [2Prop-CN, 3But-CN, 3-hydroxypentenenitrile (2OH3But-CN), 4-(methylthio)butanenitrile (3MTP-CN), 5-(methylthio)pentanenitrile (4MTB-CN), 4-(methylsulfinyl)butanenitrile (3MSOP-CN), 5-(methylsulfinyl)pentanenitrile (4MSOB-CN), 2PE-CN, IAN, 4-methoxy-3-indoleacetonitrile (4MOIAN), and 1-methoxy-3-indoleacetonitrile (1MOIAN)], seven ITCs [2Prop-ITC, 3But-ITC, 3MTP-ITC, 4MTB-ITC, 3MSOP-ITC, 4MSOB-ITC, and 2PE-ITC], the ITC derivates oxazolidine-2-thione (OZT), and 1-methoxyindol-3-carbinol (1MOI3C), and three EPTs [CETP, 1-cyano-3,4-epithiobutane (CETB), 1-cyano-2-hydroxy-3,4-epithiobutane (CHETB)]. Sprouts always formed higher GLS hydrolysis product concentrations compared to the developmental stage of fully developed heads (**Figures 2C–6C**). Among the *B. oleracea* varieties analyzed, sprouts of the savoy cabbage cv. Daphne formed the highest concentration of total hydrolysis products with 7.39 μmol/g FW (**Figure 6C**), while among the fully developed vegetables, cauliflower heads of cv. Momentum formed the lowest concentration of total hydrolysis products (0.12 μmol/g FW) (**Figure 3D**). Usually nitriles or EPTs dominated both in sprouts and the fully developed *B. oleracea* heads (**Figures 2C,D–6C,D**). However, sprouts of the red cabbage cv. Integro (**Figure 5C**), broccoli sprouts cv. Sirtaki (in 2015) (Supplementary Figures 1C) and also most of the cabbage sprouts in 2015 formed mainly ITCs (Supplementary Figures 3C–5C). Hydrolysis products of indole GLS were only detected in very low amounts and among them IAN usually was the main indole hydrolysis product detected (Supplementary Table 3).

#### Broccoli

Among the different broccoli cultivars, broccoli sprouts cv. Sirtaki showed the highest total concentration of GLS hydrolysis products (2.90 μmol/g FW), but only in 2014 (**Figure 2C**). Contrary in 2015, only nearly half of this amount of GLS hydrolysis products was formed. Moreover, the fully developed heads of this cultivar formed the lowest total level of GLS hydrolysis products (**Figure 2D**). In general, broccoli sprouts released 3–4 times more hydrolysis products compared to the fully developed heads. The main hydrolysis product detected in both developmental stages usually was 4MSOB-CN with up to 1.26 μmol/g FW in cv. Sirtaki sprouts. Nitriles, such as 4MSOB-CN, accounted for 48–79% of total hydrolysis products in sprouts and 35–65% in the fully developed heads. Next to 4MSOB-CN, the corresponding 4-MSOB-ITC was also formed in considerable amounts ranging from 0.27 μmol/g FW (cv. Marathon) to 1.18 μmol/g FW (cv. Sirtaki) in broccoli sprouts and from 0.065 μmol/g FW (cv. Sirtaki) to 0.16 μmol/g FW (cv. Iron Man) in the fully developed heads (**Figures 2C,D**). In addition to the hydrolysis products of 4MSOB, 3MSOP-CN was found in hydrolyzed sprouts cvs. Iron Man and Marathon (up to 0.40 μmol/g FW in cv. Iron Man), but not in cv. Sirtaki, whereas the corresponding 3MSOP-ITC was typically detected in lower concentrations in these three cultivars (**Figure 2C**). In the fully developed heads, 3MSOP-CN and 3MSOP-ITC were also present (**Figure 2D**), while cv. Sirtaki also formed much CHETB (up to 0.13 μmol/g FW in 2015). Finally, hydrolyzed broccoli sprouts from all three cultivars had also considerable levels of 4MTB-CN, while higher concentrations of 4MTB-ITC were only found in cv. Sirtaki sprouts (up to 0.26 μmol/g FW) (**Figure 2C**).

#### Cauliflower

Cauliflower sprouts also formed much higher concentrations in total GLS hydrolysis products compared to fully developed heads. Hydrolyzed sprouts were determined with levels up to 5.53 μmol/g FW (cv. Baltimore) as well as being 6-times (cv. Graffiti) to 35-times (cv. Momentum) richer in hydrolysis products compared to the homogenized fully developed heads (**Figures 3C,D**). The main hydrolysis product from sprouts was CETP with up to 4.40 μmol/g FW (cv. Baltimore). EPTs accounted for 65–80% of hydrolysis products in homogenized sprouts. In addition to CEPT, the nitriles 2Prop-CN, 3MTP-CN, and 3MSOP-CN were also present in notable concentrations, while 3MSOP-ITC was only present in minor concentrations. In the fully developed heads, the hydrolysis product profile differed compared to the sprouts, and CETP was only one of the many hydrolysis products ranging from 0.028 μmol/g FW (cv. Momentum) to 0.14 μmol/g FW (cv. Graffiti) (**Figure 3D**). The nitriles 3MTP-CN and 2Prop-CN and also 3MSOP-CN (especially in 2015) were main hydrolysis products and nitriles accounted for 34–75% of the total hydrolysis products of the fully developed heads. Especially in cv. Graffiti, ITCs accounted for up to 48% of all hydrolysis products, and ITCs, such as 2Prop-ITC and 3MSOP-ITC, were formed with up to 0.062 and 0.26 μmol/g FW in this cultivar, respectively (**Figure 3D**).

#### White Cabbage

White cabbage sprouts formed 4- to 8-times more GLS hydrolysis products compared to fully developed heads with cv. Tolsma sprouts having the highest concentrations (6.49 μmol/g FW) (**Figure 4C**). With one exception (cv. Perfecta sprouts in 2015), CETP was the main hydrolysis product formed both in homogenized white cabbage sprouts (**Figure 4C**) and in fully developed heads (**Figure 4D**). EPTs accounted for 54–89% of all hydrolysis products in fully developed cabbage heads. Cv. Tolsma was richest in CETP and formed up to 5.73 and 0.76 μmol/g FW in sprouts and fully developed heads, respectively. In sprouts of all cultivars, next to this EPT, 2Prop-CN and in cv. Perfecta, 3MSOP-CN and 3-MSOP-ITC were formed, but in distinctly lower amounts (**Figure 4C**). In 2015, high concentrations of the corresponding 2Prop-ITC were released (up to 1.75 μmol/g FW in cv. Tolsma sprouts) and 3MSOP-ITC was the major hydrolysis product in cv. Perfecta sprouts (1.28 μmol/g FW) (Supplementary Figure 3C). In the fully developed heads, next to CETP, like in sprouts, 2Prop-CN was formed (**Figure 4D**). In 2015, 3MSOP-CN was formed in higher concentrations in fully developed heads of cvs. Marcello and Perfecta (Supplementary Figure 3D). Finally, ITCs accounted only for 1.9% (cv. Tolsma 2015) to 23% (cv. Perfecta 2014) of all hydrolysis products.

#### Red Cabbage

Homogenized sprouts of red cabbage contained 2- to 8-times more total GLS hydrolysis products compared to fully developed heads (**Figures 5C,D**). While in 2014, sprouts of cv. Roodkop 2 formed most hydrolysis products (5.83 μmol/g FW), in 2015, cv. Redma was richest in hydrolysis products (4.08 μmol/g FW) (**Figure 5C** and Supplementary Figure 4C). In 2014, in the sprouts of cvs. Redma and Roodkop 2, the main hydrolysis product was CETP, and EPTs accounted for 50–53% of the hydrolysis products. However, in 2015, cv. Roodkop 2 sprouts formed mainly CHETB with 0.77 μmol/g FW. In contrast, sprouts of cv. Integro grown in 2014 formed high concentrations of 4MSOB-ITC and ITCs and EPTs accounted for 61 and 28% of the hydrolysis products, respectively (**Figure 5C**). Notably, sprouts grown in 2015 formed even more ITCs (86, 61, and 34% in cvs. Integro, Redma, and Roodkop 2, respectively) and fewer EPTs (13, 23, and 42%). Thus, in 2015, 3MSOP-ITC and 4MSOB-ITC were the main hydrolysis products in sprouts of cvs. Redma and Integro with cv. Integro also containing much 2Prop-ITC and OZT, a cyclization product of 2OH3But-ITC.

In the fully developed heads, most hydrolysis products were found in cv. Integro (**Figure 5D**), and 4MSOB-CN was, together with CETP, the major hydrolysis product formed with up to 0.55 μmol/g FW in this cultivar (2015). Other important hydrolysis products in fully developed red cabbage heads were the EPTs CETB and CHETB, and EPTs accounted for 25–60% of the GLS hydrolysis products. Further, nitriles accounted for 37–69% of all GLS hydrolysis products in fully developed red cabbage heads.

#### Savoy Cabbage

As demonstrated in the other *B. oleracea* varieties, sprouts of the savoy cabbage cultivars also formed 4- to 29-times the amount of total hydrolysis products compared to the fully developed heads. Cv. Daphne sprouts formed the highest total GLS hydrolysis concentrations (7.39 μmol/g FW), while homogenized cv. Emerald sprouts exhibited the lowest content among both sprouts and fully developed heads (**Figures 6C,D**). CETP was found to be the main hydrolysis product from sprouts and savoy cabbage heads. Among the sprouts, cv. Daphne formed the highest CETP levels (5.19 μmol/g FW), while in fully developed heads, cv. Capriccio had the highest CETP concentration (0.50 μmol/g FW). Sprouts also contained the nitriles 2Prop-CN, 3MTP-CN, and 3MSOP-CN, and in 2015, the ITCs 2Prop-ITC and 3MSOP-ITC were also detected (**Figure 6C** and Supplementary Figure 5C). In sprouts grown in 2014, EPTs accounted for 70–80% of the hydrolysis products, whereas in 2015, only 25–37% were EPTs and 37–67% were ITCs. In homogenized fully developed heads, 2Prop-CN along with 3MSOP-CN and 3MSOP-ITC were identified, while cv. Capriccio formed also much CETB and CHETB. In fully developed savoy cabbage heads, 46–71% of the hydrolysis products were EPTs.

In the Supplementary Figure 6 the PCA for the total concentrations of ITC, nitriles (CN), and EPT is given for the sprouts (Supplementary Figure 6A) and for the fully developed heads of investigated *B. oleracea* varieties and cultivars (Supplementary Figure 6B). Scores and loadings are given in Supplementary Table 4. Principle component (PC) 1 is strongly associated with EPT in *B. oleracea* sprouts and fully developed heads, explaining 67% of the variation (Supplementary Figures 6A,B), while PC 3 is positively associated to nitriles in *B. oleracea* sprouts (Supplementary Figure 6A) and in fully developed heads to ITC (Supplementary Figure 6B). While broccoli sprouts were separated from the other varieties, probably due to absence of EPT, the other *B. oleracea* variety sprouts are not clearly distinguishable from each other (Supplementary Figure 6A). The PCA of the fully developed heads showed a division between inflorescence-forming broccoli and cauliflower to the head-forming cabbages.

### Effect of Ontogeny on Head Development, Glucosinolates and Their Hydrolysis Products in *B. oleracea* Varieties

To study the effect of ontogeny on GLS and hydrolysis product formation, from each *B. oleracea* variety one cultivar, namely the broccoli cv. Iron Man, the cauliflower cv. Abeni, the white cabbage cv. Marcello, red cabbage cv. Redma, and savoy cabbage cv. Emerald was also harvested in the mini head and in the over-mature stage. Head developmental stages were classified according to the BBCH-scale (BBCH-Monograph, 1997). At the developmental stage 1 (mini head), the inflorescence vegetables broccoli cv. Iron Man and cauliflower cv. Abeni had 64% of the expected head diameter, while at developmental stage 2 (fully developed heads = commercial harvest), they had 100% of the expected head diameter, and at developmental stage 3 (over-mature stage), they showed a head diameter of 130 and 165%, respectively, of the expected head diameter. The head-forming cabbages in developmental stage 1 reached 80–82% of the expected head diameter, the developmental (=commercial) stage 2 was characterized by 100% head development, and developmental stage 3 revealed similar head diameters of over-mature cabbages. In **Table 1**, head surface curvature diameter (cm) values are given for these three developmental stages.

**Table 1:** Head surface curvature diameter (cm) at harvest of three different developmental stages 1 (mini head), 2 (fully developed), and 3 (over-mature) of cultivars of five *Brassica oleracea* varieties.

Cultivars	Developmental stage		
	1	2	3
**Broccoli**			
Iron Man	13.30 ± 2.18	20.71 ± 2.06	26.94 ± 5.88
Marathon		20.75 ± 2.35	
Sirtaki		21.20 ± 2.14	
**Cauliflower**			
Abeni	17.61 ± 2.49	27.70 ± 2.57	45.61 ± 9.50
Baltimore		21.98 ± 6.14	
Graffiti		23.72 ± 2.61	
Momentum		25.25 ± 4.49	
**White cabbage**			
Marcello	41.90 ± 3.01	50.99 ± 4.13	53.94 ± 5.40
Perfecta		54.88 ± 2.65	
Tolsma		51.37 ± 2.28	
**Red cabbage**			
Integro		47.12 ± 3.87	
Redma	40.40 ± 2.41	49.98 ± 5.85	48.40 ± 3.83
Roodkop 2		53.40 ± 5.68	
**Savoy cabbage**			
Capriccio		51.99 ± 4.04	
Daphne		55.29 ± 8.15	
Emerald	39.86 ± 7.10	49.46 ± 4.40	51.04 ± 4.69

*Values represent the mean of two experiments (2014 and 2015) with 25 replicates each. Presented are means ± standard deviations (SD).*

**FIGURE 7:**
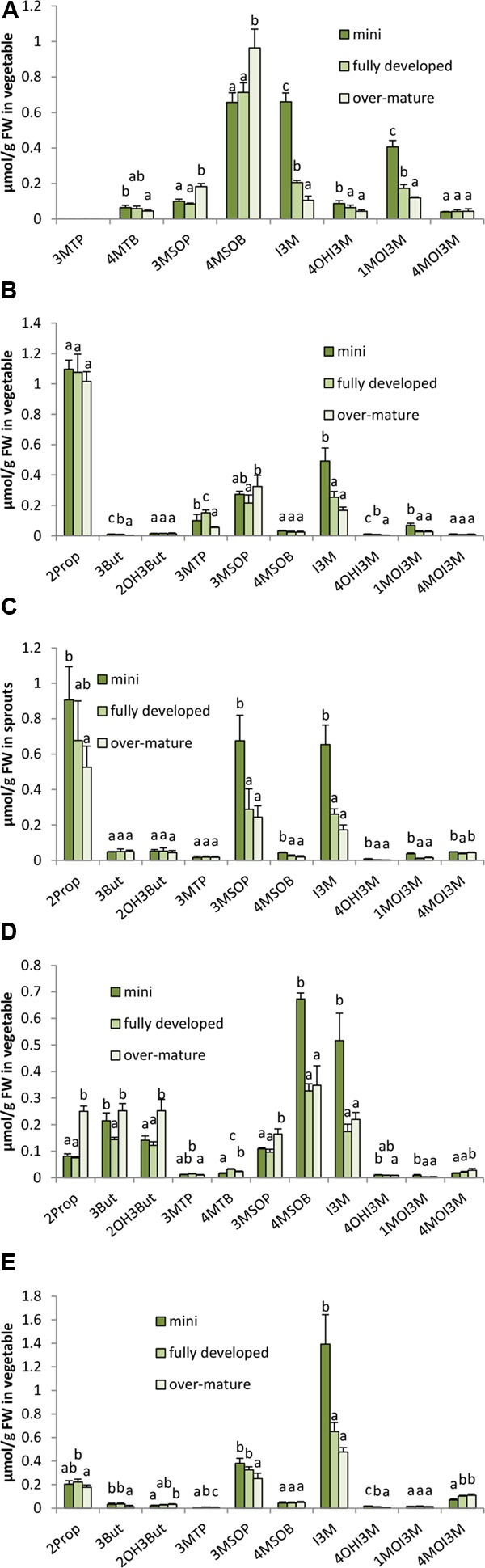
Influence of head ontogeny on the glucosinolate content [μmol/g FW] in broccoli **(A)**, cauliflower **(B)**, white cabbage **(C)**, red cabbage **(D)**, and savoy cabbage **(E)**. Abbreviations: see **Table 2**. Small letters indicate significant differences between means of glucosinolate concentrations of different head developmental stages (*p* ≤ 0.05) as determined by Tukey’s HSD test.

**FIGURE 8:**
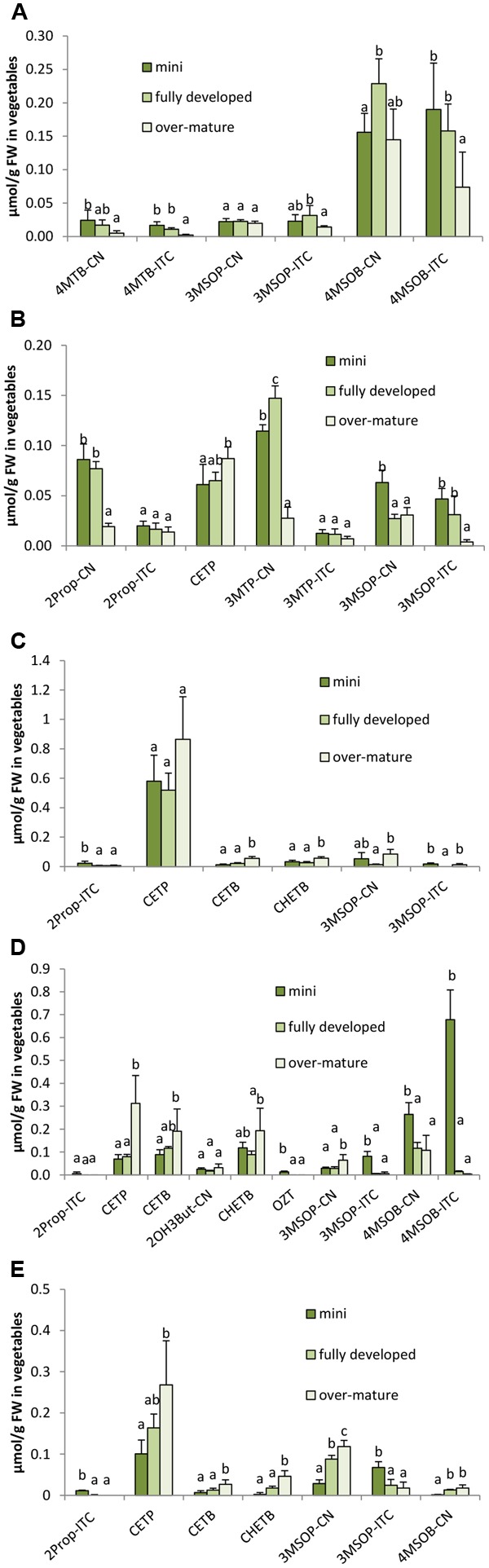
Influence of head ontogeny on the glucosinolate hydrolysis products [μmol/g FW] in broccoli **(A)**, cauliflower **(B)**, white cabbage **(C)**, red cabbage **(D)**, and savoy cabbage **(E)**. Abbreviations: see **Table 2**. Small letters indicate significant differences between means of glucosinolate hydrolysis products concentrations of different head developmental stages (*p* ≤ 0.05) as determined by Tukey’s HSD test.

In all five *B. oleracea* varieties, in 2014 significant differences between the different ontogenetic stages of head development were observed for the GLSs as well as on the hydrolysis product formation (**Figures 7, 8**). In all *B. oleracea* varieties studied in 2014, the indole GLSs, except 4MOI3M, were highest in the mini head stage and then decreased in the following stages from fully developed to over-mature heads (**Figure 7**). Similarly, the aliphatic GLSs 2Prop, 3MSOP, and 4MSOB in white cabbage cv. Marcello (**Figure 7C**), 4MSOB in the red cabbage cv. Redma (**Figure 7D**), and 3MSOP in savoy cabbage cv. Emerald were also observed to decrease in the later developmental stages (**Figure 7E**). In some cases, GLSs in 2014 also increased during development with the over-mature stage head containing most GLS as was the case for 3MSOP and 4MSOB in broccoli (**Figure 7A**), for 2Prop, (R)2OH3But, 3MSOP, and 4MOI3M in red cabbage (**Figure 7D**), and for 3MTP and 4MOI3M in savoy cabbage (**Figure 7E**). Sometimes, in 2014 GLSs were not affected by the head development, e.g., 4MOI3M in broccoli, 2Prop, 4MSOB, and (R)2OH3But in cauliflower, 3But, (R)2OH3But, and 3MTP in white cabbage, as well as 4MSOB and 1MOI3M in savoy cabbage (**Figure 7**). In 2015, for several GLS similar trends during ontogeny were found (for example I3M, 3MSOP in white and savoy cabbage), while others were differently affected (Supplementary Figure 7).

**Table 2:** Abbreviations for glucosinolates (GLS) and their derived hydrolysis products: isothiocyanates (ITC) and derivates, nitriles (CN, cyanide), and epithionitriles.

	Glucosinolates (GLS)		Corresponding breakdown products					
			Isothiocyanate (ITC)		Nitrile		Epithionitrile	
	Abbreviation	Name	Abbreviation	Name	Abbreviation	Name	Abbreviation	Name
aliphatic	2Prop	2-propenyl (or allyl) GLS	2Prop-ITC	2-propenyl (or allyl) ITC	2Prop-CN	3-butenenitrile	CETP	1-cyano-2,3-epithiopropane(or 3,4-epithiobutanenitrile)
	3But	3-butenyl GLS	3But-ITC	3-butenyl ITC	3But-CN	4-pentenenitrile	CETB	1-cyano-3,4-epithiobutane(or 4,5-epithiopentanenitrile)
	(R)2OH3But	2-(*R*)-2-hydroxy-3-butenyl GLS	OZT	5-vinyl-1,3-oxazolidine-2-thione	2OH3But-CN	3-hydroxypentene-nitrile	CHETB	1-cyano-2-hydroxy-3,4-epithiobutane (or 3-hydroxy-4,5-epithiopentanenitrile
	3MTP	3-(methylthio)propyl GLS	3MTP-ITC	3-(methylthio)-propyl ITC	3-MTP-CN	4-(methylthio)-butanenitrile	–	–
	3MSOP	3-(methylsulfinyl)propyl GLS	3MSOP-ITC	3-(methylsulfinyl)-propyl ITC	3MSOP-CN	4-(methylsulfinyl)-butanenitrile		
	4MTB	4-(methylthio)butyl GLS	4MTB-ITC	4-(methylthio)-butyl ITC	4MTB-CN	5-(methylthio)-pentanenitrile	–	–
	4MSOB	4-(methylsulfinyl)butyl GLS	4MSOB-ITC	4-(methylsulfinyl)-butyl ITC	4MSOB-CN	5-(methylsulfinyl)-pentanenitrile	–	–
indolic	1MOI3M	1-methoxyindol-3-ylmethyl GLS	1MOI3C	1-methoxyindol-3-carbinol	1MOIAN	1-methoxyindol-3-acetonitrile	–	–
	4MOI3M	4-methoxyindol-3-ylmethyl GLS	n.d.	n.d.	4MOIAN	4-methoxyindol-3-acetonitrile	–	–
	4OHI3M	4-hydroxyindol-3-ylmethyl GLS	n.d.	n.d.	n.d.	n.d.	–	–
	I3M	indol-3-ylmethyl GLS	n.d.	n.d.	IAN	3-indoleacetonitrile	–	–
aromatic	2PE	2-phenylethyl GLS	2PE-ITC	2-phenylethyl ITC	2PE-CN	3-phenylpropane-nitrile	–	–

*To facilitate understanding from which GLS the hydrolysis products are derived, nitriles were abbreviated using the cyanide (CN) name.*

While sprouts regularly had the highest concentrations in GLS hydrolysis products, the ongoing head development also strongly affected the formation of the GLS hydrolysis products. Often, ITC formation decreased with the head development and the highest levels of ITCs were found in the mini heads (**Figure 8**). For example, 4MTB-ITC and 4MSOB-ITC were highest in the homogenized mini broccoli (**Figure 8A** and Supplementary Figure 8A) ITC in the mini cauliflower heads (**Figure 8B**), 2Prop-ITC in the mini white cabbage heads (**Figure 8C**), 3MSOP-ITC and 4MSOB-ITC in mini red cabbage heads (**Figure 8D**), and 3MSOP-ITC in mini savoy cabbage heads (**Figure 8E**). In 2014, nitrile levels usually behaved like those of ITCs, i.e., they decreased with head development or were not affected at all (**Figure 8**). In 2015, however, nitriles in white, red, and savoy cabbage often increased with further head development (Supplementary Figures 8C–E) or, as observed for 3MSOP-CN in cauliflower, first decreased from mini to fully developed heads and then subsequently increased in the over-mature stage (Supplementary Figure 8B). With regard to EPT formation, overall a similar trend was observed and CETP, CETB, and/or CHETB increased from the mini to fully developed heads. This effect was significant for CETP in cauliflower (**Figure 8B**), for CETP, CETB, and CHETB in red cabbage (**Figure 8D**) and in savoy cabbage (**Figure 8E**), and for CETB in white cabbage (**Figure 8C**). Notably, broccoli heads contained no EPTs due to the lack of alkenyl GLS.

## Discussion

### Glucosinolates in *B. oleracea* Varieties

Sprouts of *B. oleracea* varieties contained up to 11-times more GLSs compared to the fully developed heads – a finding also reported by Fahey et al. (1997) and particularly observed in broccoli. In our study, the GLS profile found in the sprouts of all five analyzed *B. oleracea* varieties mainly contained aliphatic GLSs, while the fully developed heads were also rich in indole GLSs (**Figures 2A,B–6A,B**). Increased concentrations of indole GLSs of mature *B. rapa* ssp. *chinensis* leaves compared to the sprouts were also observed by Wiesner et al. (2013b), who characterized the GLS profiles of different pak choi cultivars. Probably indole GLS levels increase during early ontogeny as they are important for the plant’s protection from biotic stressors (Textor and Gershenzon, 2009).

With regard to the individual GLSs in the different *B. oleracea* varieties, we found that 4MSOB was the main GLS in broccoli sprouts and in the corresponding heads, 3MSOP or 4MTB was also present (**Figures 2A,B**). Similarly, Brown et al. (2002) also reported 4MSOB as the major GLS in fully developed broccoli heads. Notably, the authors also found high levels of (R)2OH3But in their investigated broccoli cultivars, which in the present study, was only a major GLS of broccoli cv. Sirtaki in 2015. Further, the GLS profile found in the broccoli cv. Marathon correlated well with the results reported by others for this cultivar (Rosa and Rodrigues, 2001).

With regard to cauliflower, sprouts contained mainly 2Prop and 3MSOP as was similarly reported previously (Bellostas et al., 2007), whereas the fully developed heads were also rich in I3M and had a considerable concentration of 3MTP. Interestingly, Gu et al. (2015) have also recently reported that 2Prop and I3M are the main GLSs in fully developed cauliflower heads of one cultivar, while other cultivars were mainly rich in 3MSOP. Likewise, in a study by Schonhof et al. (2004), 3MSOP was a major GLS found in cauliflower cultivars, while some of these cultivars also contained 3MTP, 4MTB, or 4MSOB as a main GLS.

Overall, in the present study, both developmental stages, namely sprouts and fully developed heads, of the white and savoy cabbage cultivars were dominated by 2Prop, 3MSOP, and I3M, reconfirming previous *Brassica* studies (Ciska et al., 2000; Bellostas et al., 2007; Hirono et al., 2011). Generally, sprouts of the three red cabbage cultivars contained mainly 4MSOB, but also had considerable levels of 2Prop, (R)2OH3But, and 3MSOP. In contrast, Bellostas et al. (2007) found much more (R)2OH3But in red cabbage sprouts (cv. Debut) than observed in the present study. In our study, fully developed red cabbage heads were also rich in 3But and I3M, which correlates well with former reports (Ciska et al., 2000; Oerlemans et al., 2006).

In many cases, the GLS profiles of sprouts and corresponding fully developed vegetables of the present study correlated well between the 2 years (**Figures 2–6** and Supplementary Figures 1–5). In other cases the profiles varied considerably. Differences in profiles and concentrations between the years, such as observed for the cauliflower cv. Abeni that had much more 2Prop in 2014 or for red cabbage cv. Roodkop 2 sprouts that had much more (R)2OHBut and 4MSOB in 2015 compared to 2014 (**Figure 5A** and Supplementary Figure 4A), can be caused by changed environmental conditions, such as climatic factors, e.g., radiation intensity and duration as well as temperature (Schonhof et al., 2007; Schreiner et al., 2009). For example, broccoli grown at 18°C with a 24 h light regime had a lower 4MSOB concentration than broccoli grown at 12°C under the same light regime. On the other hand, 3MSOP, 4OHI3M, and 1MOI3M concentrations were increased in broccoli grown at 18°C compared to 12°C with a 12 h photoperiod (Mølmann et al., 2015). Thus, we hypothesize that the lower temperature or higher radiation in 2015 (Supplementary Figure 9) compared to 2014 led to induced aliphatic GLS synthesis: in the cv. Roodkop 2 sprouts probably methylthioalkylmalate synthases (MAM) catalyzed side-chain elongation resulting in the synthesis of more 4MSOB and via 2-oxoglutarate-dependent dioxygenase AOP3 mediated catalysis (R)2OH3But was formed (Sønderby et al., 2010). In mature cv. Abeni it is likely that AOP2 was increased in 2014, which transformed 3MSOP to 2Prop (Sønderby et al., 2010).

### Glucosinolates Hydrolysis Products in *B. oleracea* Varieties

Upon GLS hydrolysis in the *B. oleracea* varieties studied, in both sprouts and fully developed heads nitriles and EPTs were often mainly formed instead of ITCs (**Figures 2C,D–6C,D**). That nitriles in general and EPTs in particular can be the major hydrolysis products of *Brassica* GLSs has been also observed in earlier studies (Cole, 1975; Kyung et al., 1995; Hanschen et al., 2015) and is caused by the presence of ESP enzymes (Matusheski et al., 2006).

With regard to ITC formation, the cancer-preventive 4MSOB-ITC was mainly formed in homogenized sprouts of the broccoli cv. Sirtaki with up to 1.2 μmol/g FW. However, 1.3 μmol/g FW of the corresponding nitrile was also present. In broccoli, the formation of aliphatic nitriles, such as 4MSOB-CN, is caused by the ESP that catalyzes the formation of nitriles from the non-alkenyl GLS-aglucone (Mithen et al., 2003; Matusheski et al., 2004).

While 4MSOB-ITC is known as a cancer-preventing agent, the corresponding nitrile was reported to be a much less potent inducer of phase-II enzymes (Matusheski and Jeffery, 2001). Moreover, using a liver cancer cell (HepG2 cells) model, our group has recently shown that nitriles are less cytotoxic compared to ITCs; however, like ITCs, nitriles do have genotoxic potential that is further increased by *CYP2E1* overexpression (Kupke et al., 2016). Further, in the *B. oleracea* varieties studied here, CETP was one of the most abundant hydrolysis products found especially in sprouts of cauliflower, white, red, and savoy cabbage as well as in fully developed white cabbage and savoy cabbage heads. Of note is that concentrations of up to 5.7 μmol or 0.56 mg/g FW (=77.6 μmol or 7.7 mg/g DW) in white cabbage sprouts (cv. Tolsma) and up to 0.8 μmol/g FW (=9.12 μmol or 0.90 mg/g DW) in the correspondingly fully developed heads were found. Consistent with our observations, other groups also identified CETP as the main GLS hydrolysis product in white cabbage but Rungapamestry et al. found only up to 0.2 μmol/g DW (3% recovery of initial 2Prop level) (Kyung et al., 1995; Rungapamestry et al., 2006). In addition to CETP, CHETB has also been found in cabbage heads which is also in line with our study (Daxenbichler et al., 1977). Moreover, Cole (1980) reported 8.7 μg/g FW (0.087 μmol/g FW) CETP in 12-week-old cauliflower, which corresponds well to our results (0.03–0.14 μmol/g FW). While ITCs have been shown to exert pleiotropic effects against cancer (Lamy et al., 2011; Veeranki et al., 2015), CETP was shown to affect cancer and healthy cells in a non-selective way by inducing cell death of HepG2 cells and primary mouse hepatocytes by necrosis (Hanschen et al., 2015). However, CETP was also reported to have the potential to induce of phase-II enzymes in rat liver RL-34 cells (Kelleher et al., 2009).

Concerning the recovery of the hydrolysis products in the homogenized sprouts and fully developed heads it was often lower than 100% and varied among the cultivars. Indole hydrolysis products such as IAN always had a very low recovery (Supplementary Table 3). Probably this is linked to the high reactivity of the indole ITCs and carbinols that react to non-volatile compounds such as ascorbigen (Agerbirk et al., 2009) and thus were not detected with the current GC method. The recovery of the aliphatic GLS usually was much higher. However, while for example in fully developed cauliflower cv. Graffiti the recovery of the GLS hydrolysis products was high, the other cauliflowers cultivars formed much less hydrolysis products (**Figure 3** and Supplementary Figure 2). It is likely that these cultivars had a decreased myrosinase activity and therefore less hydrolysis products were recovered. Decreased myrosinase activity probably was also the reason for the low levels of hydrolysis products formed in broccoli cv. Sirtaki in 2015 compared to 2014 (only 44% compared to 2014), while 70% of the GLS concentration of 2014 was present. As ITC are reactive electrophilic compounds, another reason for a decreased recovery could also be the reaction of ITC with nucleophiles such as cysteine, glutathione or other thiols to non-volatile products that occurs also under acidic conditions (Hanschen et al., 2012). As the concentration of glutathione in *A. thaliana* is around 0.225 μmol/g FW (Meyer et al., 2007) it is likely that these reactions could occur in all Brassicaceae plants. Moreover, a low recovery in sulfinyl hydrolysis products such as 3MSOP-CN or -ITC can be also caused by a dirty GC-liner, if not changed very regularly as performed in our study.

With regard to differences in GLS hydrolysis product profiles between the 2 years studied, most of the cabbage sprouts grown in summer 2015 (white, red, and savoy cabbages) had far higher ITC concentrations compared to the cabbage sprouts grown in summer 2014 (**Figures 2–6** and Supplementary Figures 1–5). These changes observed for the GLS hydrolysis products may be due to decreased ESP activities in the sprouts of 2015 compared to 2014, since ESP protein catalyzes the formation of EPTs and nitriles (Matusheski et al., 2006; Wittstock and Burow, 2010). To the best of our knowledge, it is currently not known which abiotic or biotic factors affect ESP activity. However, a decrement of ESP transcripts was reported after UV-B radiation treatment, thereby indicating that environmental factors affect hydrolysis (Mewis et al., 2012). In our study, when sprouts were grown (summer 2014/2015) slight differences in climatic factors, such as temperature, were recorded. For example, during the week of sprout growth, it was in the first 4 days of sprout growth on average 4°C warmer in 2014 compared to 2015 and then it was in average 4°C colder in 2014 compared to 2015 (Supplementary Figure 9). Thus, differences in temperature might be responsible for the higher ITC content.

### Effect of Ontogeny on Glucosinolates and Their Hydrolysis Products in *B. oleracea* Varieties

With regard to the effect of ontogeny on the formation of GLSs and their respective hydrolysis products in broccoli, cauliflower, as well as white, red, and savoy cabbage, a variety and structure-specific response both for GLSs as well as for their hydrolysis products was observed (**Figures 7, 8**). For example, the alkenyl GLSs, namely 2Prop, 3But, and (R)2OH3But, but also the sulfinyl 3MSOP were increased in over-mature red cabbage, while the methylsulfinylalkyl GLS 4MSOB and the indole GLS I3M were decreased. Probably *de novo* synthesis of 3MSOP occurred, but with decreased MAM catalyzed side-chain elongation less 4MSOB would be formed. Further, an increased AOP2 and AOP3 activity would explain the formation of the alkenyl GLS (Sønderby et al., 2010). In white cabbage, 2Prop as well as the methylsulfinylalkyl GLSs 3MSOP and I3M decreased during head development (**Figure 7**). The observed reduction of I3M in all *B. oleracea* vegetables studied here probably is due to a dilution effect of the GLS during plant growth in the absence of biotic stressors, as herbivory would strongly increase the tryptophan dependent indole GLS biosynthesis (Textor and Gershenzon, 2009).

When studying the effect of ontogeny during the complete life cycle of broccoli, in contrast to our results of 2014, Rangkadilok et al. (2002) observed a continuous decrement in the methylsulfinylalkyl GLS 4MSOB concentrations during ontogeny from seeds via sprouts, the vegetative stage, and green head to flowering stage, while Vallejo et al. (2003) reported in broccoli cv. ‘Marathon’ and ‘Monterrey’ in general an increment of 4MSOB during head development after poor sulfur fertilization (15 kg/ha CaSO_4_) which is similar to our study (no sulfur fertilization). While there are several studies on the ontogenetic effect of *B. oleracea* head development on the GLS profile in *B. oleracea*, less is known about the effect on GLS hydrolysis products. In the present study, ITCs usually decreased from the mini head stage to the over-mature stage (**Figure 8**), whereas EPT formation often increased during head development, even when the respective GLS was not affected. These observations suggest that changes in ESP activity or other factors affecting hydrolysis occur due to ontogeny. For example also a change in myrosinase activity or glucosinolate concentration might affect the hydrolysis outcome even if ESP activity is not affected. For example it was shown, that by adding inactivated plant material (which still contained GLS) or by adding water, ITC formation during hydrolysis increased, as probably the probability of the ESP protein to encounter the aglucone, formed by myrosinase degradation, will decrease (Hanschen et al., 2017). Moreover, also the vitamin C concentration can affect the hydrolysis as it is a cofactor of myrosinases and a high myrosinase activity would favor ITC formation (Burow et al., 2006). Fe^2+^ on the other hand is a cofactor for ESP and therefore enhances its activity, that is also substrate specific (Burow et al., 2006) (**Figure 1**). Nevertheless, the ITC concentration also will increase if more GLS are present. For example, in the present study 4MSOB in broccoli increased during head ontogeny (**Figure 7A**) while total 4MSOB breakdowns and particularly 4MSOB-ITC decreased (**Figure 8A**). This could be explained by a reduction in myrosinase activity or an increase in compounds that would mask ITC such as glutathione, as thiols are highly reactive with ITC (Hanschen et al., 2012).

During the initial sprout growth phase in broccoli, ESP activity was shown to decrease and ITC formation to increase (Williams et al., 2008). In contrast, in further developmental stages, similar results to our study were reported. For example, in cauliflower, 2Prop-ITC declined between 9 and 16 weeks of growth, while CETP increased from 10 to 12 weeks of growth and then decreased thereafter (Cole, 1980). In watercress (*Nasturtium officinale*), 2-phenylethyl ITC increased in leaves starting from 21-day-old plants for the next 40 days and then remained unchanged (Palaniswamy et al., 2003). Thus, plant ontogeny not only has an impact on GLS formation in the plants, but also on the formation of the hydrolysis products. In addition to the ontogenetic effect on both GLS biosynthesis and degradation, the ontogenetic effect seems to be dependent on the *Brassica* species as well as on the plant organ.

In summary, *B. oleracea* varieties are an important GLS source for the human diet. While broccoli and red cabbage exhibited high levels of 4MSOB, cauliflower, as well as white and savoy cabbages were rich in 2Prop and 3MSOP. Upon hydrolysis, however, these *B. oleracea* varieties often released EPTs and nitriles instead of the protective ITCs. Thus, in order to generate vegetables rich in ITC, the selection of ITC rich cultivars is recommended, e.g., for fully developed vegetables the broccoli cv. Iron Man and cauliflower cv. Graffiti or for sprouts the broccoli cv. Sirtaki. This generates the basis for further studies on ecophysiological or elicitor induced modifications of the targeted GLS hydrolysis product formation to develop pre- and post-harvest strategies. These strategies are required in order to decrease ESP activity (or to increase myrosinase activity) in these *Brassica* vegetables, and thereby, increase health-promoting ITC formation. UVB-treatment can induce GLS formation, such as 4MSOB, and also might be a possible option for ESP gene downregulation, and thus, ITC optimization, since transcripts of an ESP-similar protein were negatively correlated to UVB treatment (Mewis et al., 2012). Moreover, the vitamin C concentration increased in UVB treated broccoli (Topcu et al., 2015), which might increase myrosinase activity as well. Further ITC formation can be also optimized due to the preparation conditions (Hanschen et al., 2017). Finally, this study has highlighted that mini heads contain far higher levels of ITCs compared to mature heads. Thus, we propose that their consumption would be more beneficial to human health from a cancer-preventive point of view.

## Author Contributions

FH and MS designed the study. FH performed the experiments. FH analyzed the data. MS contributed reagents/materials/analysis tools. FH and MS wrote the manuscript.

## Conflict of Interest Statement

The authors declare that the research was conducted in the absence of any commercial or financial relationships that could be construed as a potential conflict of interest. The reviewer RK and handling editor declared their shared affiliation, and the handling editor states that the process met the standards of a fair and objective review.
