# Supplementary material for: Isothiocyanates, Nitriles, and Epithionitriles from Glucosinolates Are Affected by Genotype and Developmental Stage in Brassica oleracea Varieties
Source: Front Plant Sci. 2017 Jun 22;8:1095. doi: 10.3389/fpls.2017.01095 (PMC5479884; doi:10.3389/fpls.2017.01095)
Supplement: Supplementary file 1 [file Table_1.DOCX]

Supplementary Table 1: Harvest dates in 2014 and 2015.

| 2014 | Broccoli | | | Cauliflower | | | | White cabbage | | | Savoy cabbage | | | Red cabbage | | |
| --- | --- | --- | --- | --- | --- | --- | --- | --- | --- | --- | --- | --- | --- | --- | --- | --- |
|  | Iron Man | Marathon | Sirtaki | Momentum | Abeni | Baltimore | Graffiti | Tolsma | Perfecta | Marcello | Emerald | Daphne | Capriccio | Redma | Integro | Roodkop 2 |
| Sowing | 18.03. | 18.03. | 18.03. | 18.03. | 18.03. | 18.03. | 18.03. | 18.06. | 18.06. | 18.06. | 18.06. | 18.06. | 18.06. | 18.06. | 18.06. | 18.06. |
| Harvest sprouts | 25.03. | 25.03. | 25.03. | 25.03. | 25.03. | 25.03. | 25.03. | 27.06. | 27.06. | 27.06. | 27.06. | 27.06. | 27.06. | 27.06. | 27.06. | 27.06. |
| Harvest mini vegetable | 11.06. |  |  |  | 11.06. |  |  |  |  | 03.09. | 03.09. |  |  | 08.09. |  |  |
| Harvest fully mature vegetable | 17.06. | 17.06. | 11.06. | 11.06. | 17.06. | 17.06. | 19.06. | 01.10. | 01.10. | 01.10. | 20.10. | 08.09. | 20.10. | 20.10. | 20.10. | 20.10. |
| Harvest over maturity stage | 26.06. |  |  |  | 26.06. |  |  |  |  | 26.11. | 26.11. |  |  | 26.11. |  |  |
|  |  |  |  |  |  |  |  |  |  |  |  |  |  |  |  |  |
| 2015 | Broccoli | | | Cauliflower | | | | White cabbage | | | Savoy cabbage | | | Red cabbage | | |
|  | Iron Man | Marathon | Sirtaki | Momentum | Abeni | Baltimore | Graffiti | Tolsma | Perfecta | Marcello | Emerald | Daphne | Capriccio | Redma | Integro | Roodkop 2 |
| Sowing | 31.03. | 31.03. | 31.03. | 31.03. | 31.03. | 31.03. | 31.03. | 22.06. | 22.06. | 22.06. | 22.06. | 22.06. | 22.06. | 22.06. | 22.06. | 22.06. |
| Harvest sprouts | 08.04. | 08.04. | 08.04. | 08.04. | 08.04. | 08.04. | 08.04. | 29.06. | 29.06. | 29.06. | 29.06. | 29.06. | 29.06. | 29.06. | 29.06. | 29.06. |
| Harvest mini vegetable | 23.06 |  |  |  | 23.06. |  |  |  |  | 10.09. | 09.09. |  |  | 16.09. |  |  |
| Harvest fully mature vegetable | 30.06. | 30.06. | 30.06. | 18.06. | 26.06. | 18.06. | 07.07. | 14.10. | 21.10. | 14.10. | 30.09. | 09.09. | 30.09. | 15.10. | 15.10. | 28.10. |
| Harvest over maturity stage | 06.07. |  |  |  | 06.07. |  |  |  |  | 12.11. | 21.10. |  |  | 12.11. |  |  |
