# Supplementary material for: Isothiocyanates, Nitriles, and Epithionitriles from Glucosinolates Are Affected by Genotype and Developmental Stage in Brassica oleracea Varieties
Source: Front Plant Sci. 2017 Jun 22;8:1095. doi: 10.3389/fpls.2017.01095 (PMC5479884; doi:10.3389/fpls.2017.01095)
Supplement: Supplementary file 2 [file Table_2.DOCX]

Supplementary Table 2: Climatic data of field experiments. Temperatures, radiation and relative humidity are given as averages.

|  | Temperature belowground  (-20 cm) (°C) | | Temperature aboveground  (+ 20 cm) (°C) | | Radiation (W/m^2^) | | Relative humidity (%) | |
| --- | --- | --- | --- | --- | --- | --- | --- | --- |
|  | 2014 | 2015 | 2014 | 2015 | 2014 | 2015 | 2014 | 2015 |
| March | 6.5 | 5.2 | 6.7 | 5.4 | 105.6 | 98.9 | 86.4 | 84.7 |
| April | 12.2 | 9.1 | 11.7 | 8.8 | 164.2 | 168.9 | 81.4 | 79.4 |
| May | 15.7 | 13.4 | 14.2 | 13.2 | 181.8 | 208.5 | 81.3 | 77.0 |
| June | 19.6 | 18.0 | 17.8 | 17.1 | 233.1 | 236.0 | 83.8 | 75.4 |
| July | 22.0 | 21.4 | 22.0 | 21.0 | 224.1 | 243.2 | 88.3 | 83.0 |
| August | 20.4 | 22.3 | 17.8 | 22.0 | 187.1 | 207.9 | 87.5 | 78.3 |
| September | 17.6 | 16.4 | 16.3 | 14.7 | 122.2 | 134.5 | 85.0 | 87.1 |
| October | 13.5 | 10.3 | 12.1 | 8.3 | 69.5 | 73.4 | 90.5 | 92.5 |
| November | 8.4 | 8.0 | 7.1 | 7.3 | 28.3 | 32.6 | 94.1 | 95.7 |
