# Supplementary material for: Isothiocyanates, Nitriles, and Epithionitriles from Glucosinolates Are Affected by Genotype and Developmental Stage in Brassica oleracea Varieties
Source: Front Plant Sci. 2017 Jun 22;8:1095. doi: 10.3389/fpls.2017.01095 (PMC5479884; doi:10.3389/fpls.2017.01095)
Supplement: Supplementary file 9 [file Image_5.PDF]

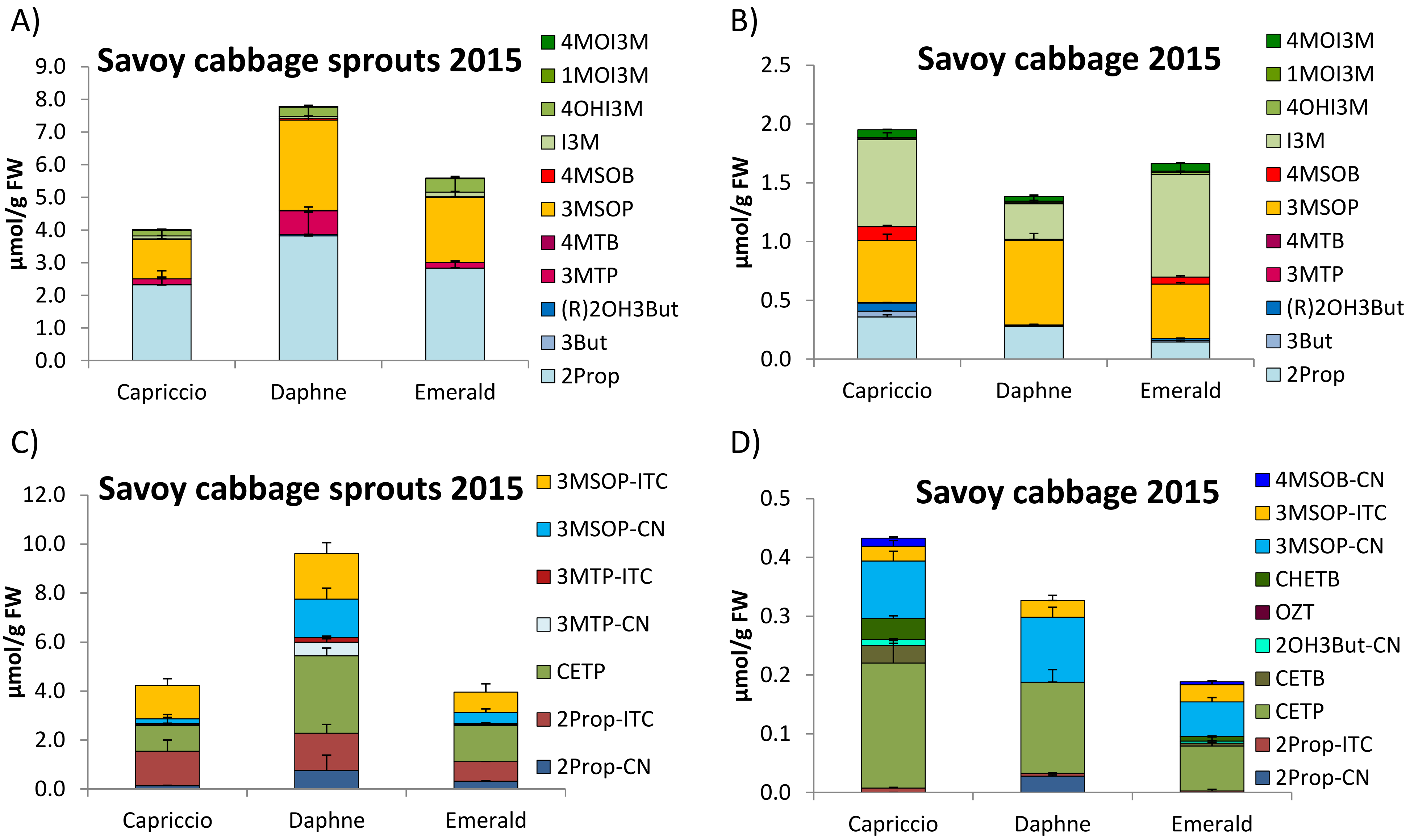


**Supplementary Figure 5**: Glucosinolates (GLSs) [µmol/g FW] and their hydrolysis products [µmol/g FW] in different cultivars of savoy cabbage in sprouts [A) GLSs, C) hydrolysis products] and fully developed savoy cabbage heads [B) GLSs, D) hydrolysis products] in 2015. Abbreviations: see Table 2.
