# Supplementary material for: Isothiocyanates, Nitriles, and Epithionitriles from Glucosinolates Are Affected by Genotype and Developmental Stage in Brassica oleracea Varieties
Source: Front Plant Sci. 2017 Jun 22;8:1095. doi: 10.3389/fpls.2017.01095 (PMC5479884; doi:10.3389/fpls.2017.01095)
Supplement: Supplementary file 10 [file Image_6.PDF]

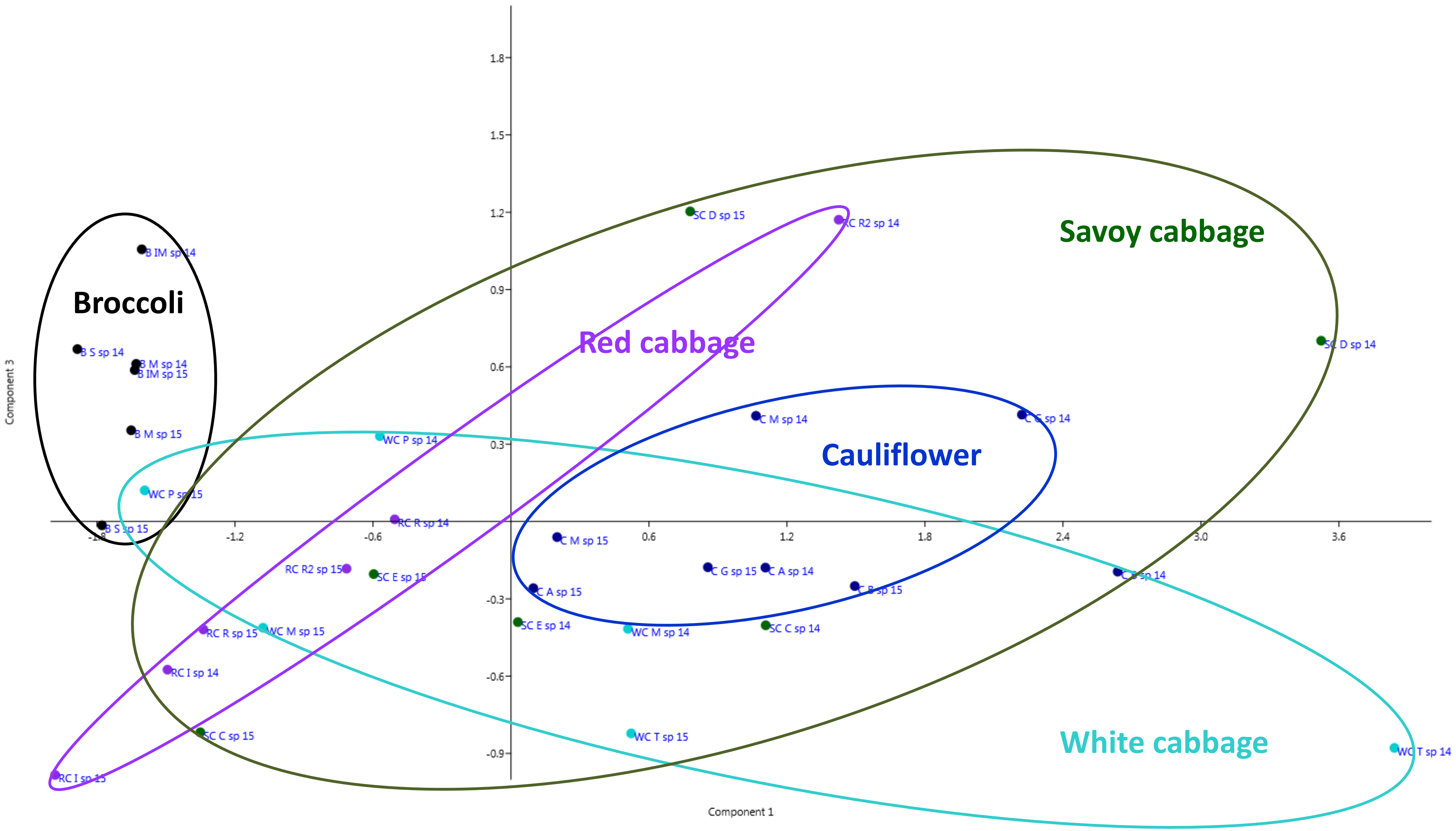

Supplemantary Figure 6A: Principle component analysis (PCA) of *B. oleracea* sprouts in 2014 and 2015.

B= Broccoli (IM- Iron Man, M- Marathon, S- Sirtaki), C= cauliflower (A- Abeni, B- Baltimore, G-Graffiti, M- Momentum), WC= white cabbage (M- Marcello, P- Perfecta, T-Tolsma), RC= red cabbage (I- Integro, R- Redma, R2-Roodkop), SC= savoy cabbage (C- Capriccio, D- Daphne, E- Emerald), sp= sprouts, 14= 2014, 15= 2015.

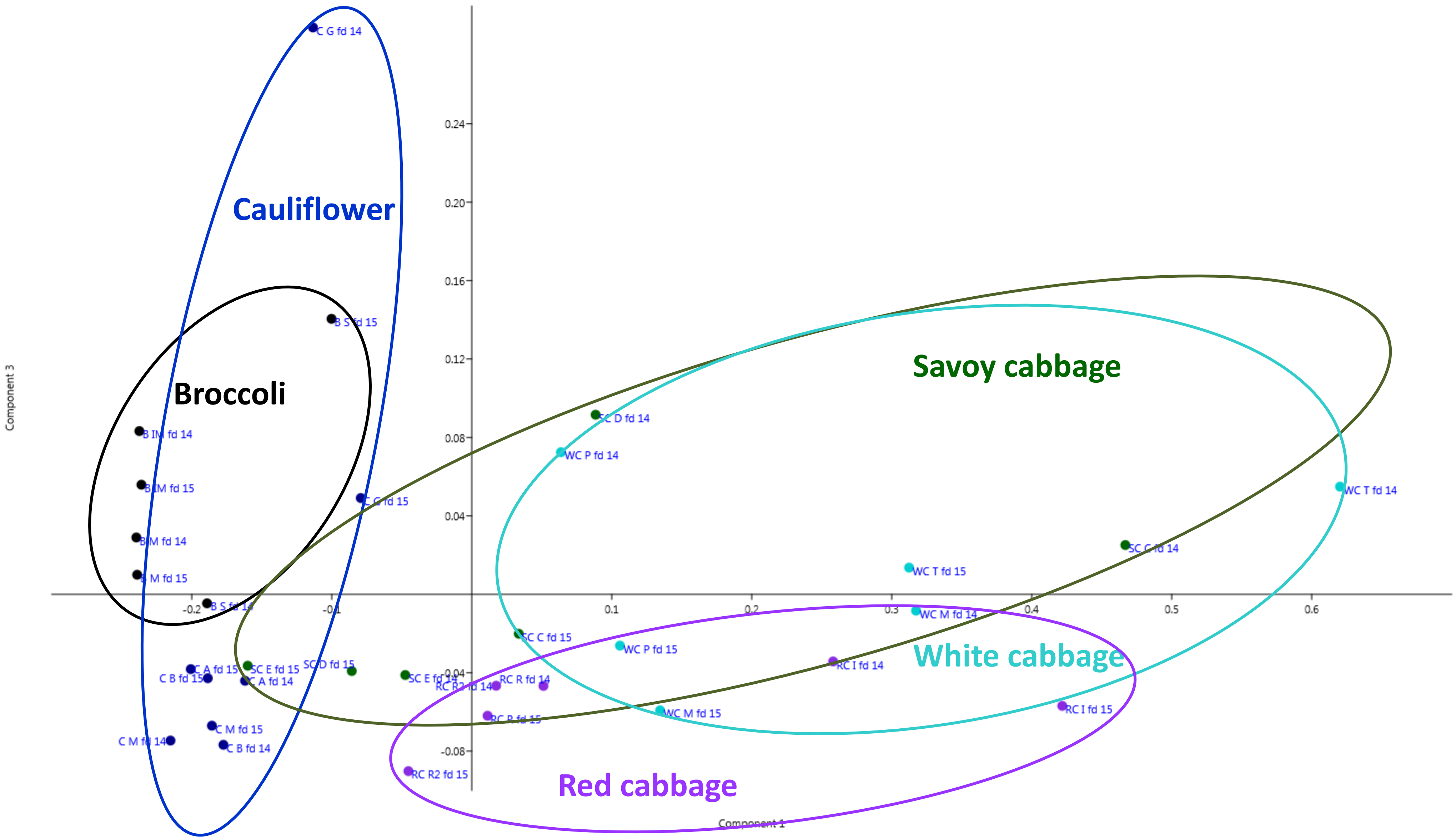

Supplemantary Figure 6B: Principle component analysis (PCA) of *B. oleracea* fully developed vegetables in 2014 and 2015.
B= Broccoli (IM- Iron Man, M- Marathon, S- Sirtaki), C= cauliflower (A- Abeni, B- Baltimore, G-Graffiti, M- Momentum), WC= white cabbage (M- Marcello, P- Perfecta, T-Tolsma), RC= red cabbage (I- Integro, R- Redma, R2-Roodkop), SC= savoy cabbage (C- Capriccio, D- Daphne, E- Emerald), fd= fully developed, 14= 2014, 15= 2015.
