# Supplementary material for: Isothiocyanates, Nitriles, and Epithionitriles from Glucosinolates Are Affected by Genotype and Developmental Stage in Brassica oleracea Varieties
Source: Front Plant Sci. 2017 Jun 22;8:1095. doi: 10.3389/fpls.2017.01095 (PMC5479884; doi:10.3389/fpls.2017.01095)
Supplement: Supplementary file 11 [file Image_7.pdf]

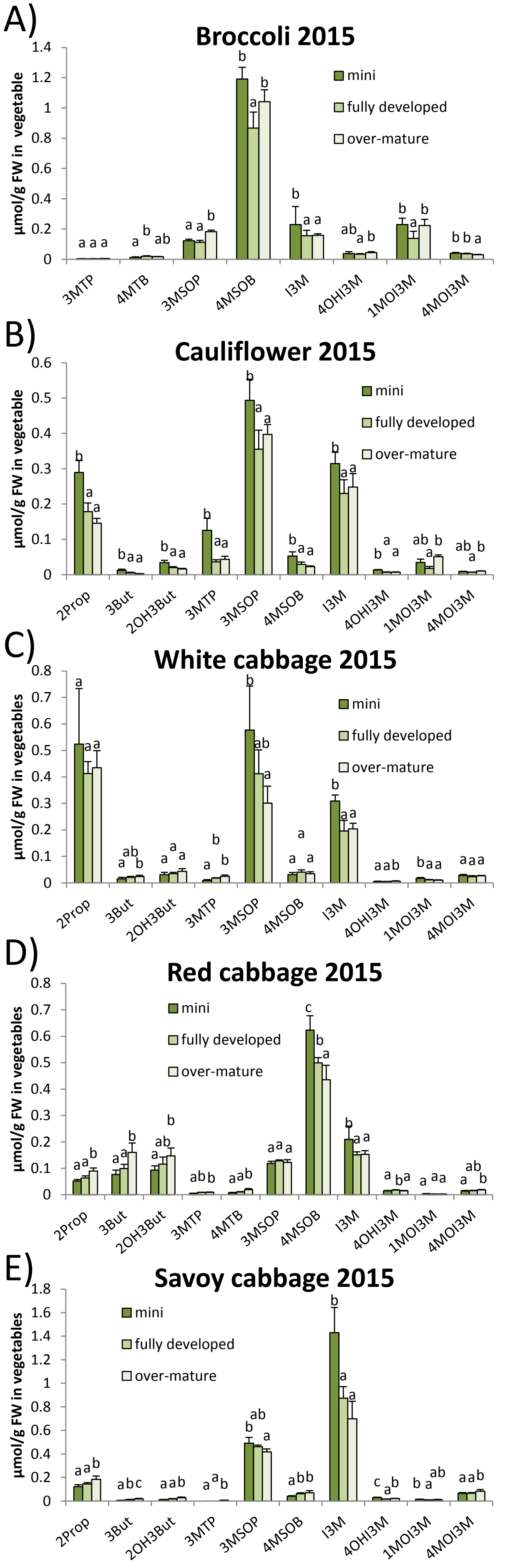

Supplementary Figure 7: Influence of head ontogeny on the glucosinolate content in 2015 [μmol/g FW] in broccoli A), cauliflower B), white cabbage C), red cabbage D), and savoy cabbage E). Abbreviations: see Table 2. Statistical information according to Figure 7.
